# Membrane Thickness Dependence of Nanopore Formation with a Focused Helium Ion Beam

**DOI:** 10.3390/s140508150

**Published:** 2014-05-06

**Authors:** Furat Sawafta, Autumn T. Carlsen, Adam R. Hall

**Affiliations:** 1 Joint School of Nanoscience and Nanoengineering, University of North Carolina Greensboro, Greensboro, NC 27401, USA; E-Mail: fsawafta@gmail.com; 2 Department of Biomedical Engineering, Wake Forest University School of Medicine, Winston-Salem, NC 27101, USA; E-Mail: acarlsen@wakehealth.edu; 3 Comprehensive Cancer Center, Wake Forest University School of Medicine, Winston-Salem, NC 27101, USA

**Keywords:** solid-state nanopores, ultrathin nanopores, helium ion microscopy, ion milling, DNA translocation

## Abstract

Solid-state nanopores are emerging as a valuable tool for the detection and characterization of individual biomolecules. Central to their success is the realization of fabrication strategies that are both rapid and flexible in their ability to achieve diverse device dimensions. In this paper, we demonstrate the membrane thickness dependence of solid-state nanopore formation with a focused helium ion beam. We vary membrane thickness *in situ* and show that the rate of pore expansion follows a reproducible trend under all investigated membrane conditions. We show that this trend shifts to lower ion dose for thin membranes in a manner that can be described quantitatively, allowing devices of arbitrary dimension to be realized. Finally, we demonstrate that thin, small-diameter nanopores formed with our approach can be utilized for high signal-to-noise ratio resistive pulse sensing of DNA.

## 1. Introduction

Solid-state (SS-) nanopores are appealing as high-throughput systems for biomolecular detection and characterization [1,2]. In the basic detection scheme, a Si chip supporting a silicon nitride (SiN) thin film window with a single embedded nanopore is positioned between two reservoirs of electrolyte solution in a flow cell. Voltage is applied across the SiN membrane, creating a strong electric field localized at the pore and initiating a steady ionic current through it. Analytes subsequently added to the appropriate chamber experience an electrical force and translocate through the opening, resulting in characteristic changes in the measured transmembrane current. This technique, while conceptually simple, is powerful and has been utilized to study a wide range of analytes, including single- [3] and double-strand (ds-) [4,5] DNA, RNA [6], proteins [7,8], nucleoprotein filaments [9,10], and solid-state materials [11–14].

Due to the high strength of the electric field inside the nanopore, the translocation process is exceedingly fast; dsDNA of 48.5 Kbp length, for instance, threads in about 1 ms under normal conditions [15]. Such rapid threading imposes a severe limitation on the resolution of the device, limiting its effectiveness in detecting small molecules as well as small features along large molecules [9], including potentially the nucleotide sequence of genomic DNA [16]. A variety of techniques has been investigated to address this issue, such as altering the translocation dynamics artificially through manipulation of solvent conditions [17–19], the use of alternative materials [20,21] and schemes [22–25], and the introduction of low-noise, high-bandwidth amplifiers [26]. One recent approach of considerable interest is the use of so-called “ultrathin” nanopore devices [27] wherein SS-nanopores are formed in membranes only a few nanometers thick. Under this geometry, a strong increase in the signal-to-noise ratio (SNR) of transient current changes associated with electrokinetic translocation has been observed due to geometric effects [27]. Improvement in SNR allows for the measurement of subtle differences in structure between small biomolecules [28,29] and for the detection of DNA oligomers as small as 16 bp in length [27]. Control over device dimensions is therefore a key aspect of enhancing signal readout during SS-nanopore detection.

**Figure 1.**
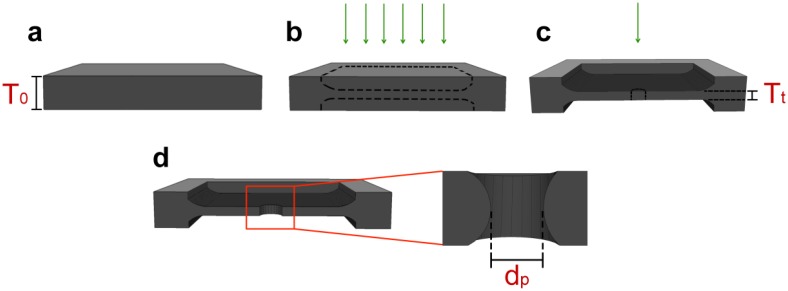
Schematic representation of ultrathin nanopore fabrication process. (**a**) Cross-section of a free-standing SiN membrane with initial thickness *T_o_*; (**b**) Patterned ion beam exposure (green arrows) of a square region inside which material is controllably removed from both sides of the membrane simultaneously, resulting in (**c**) a final thickness *T_t_*; Exposure of a single spot within the thinned region results in (**d**) a nanopore with diameter *d_p_*.

So far, the top-down fabrication of ultrathin nanopores has required lithographic patterning followed by reactive ion etching to reduce membrane thickness and subsequent resist removal before the nanopore is formed. While well-suited to batch processing, this approach is arduous for the preparation of individual devices with varying membrane thickness. A thinning mechanism that can be integrated seamlessly with an established nanopore fabrication technique would be ideal for rapid and arbitrary device preparation. While several approaches [30–36] exist for nanopore fabrication, milling with the focused beam of a scanning helium ion microscope (HIM) has been demonstrated as a uniquely effective approach to both form SS-nanopores [37] and to reduce the thickness of a suspended solid-state membrane controllably [38]. However, the effect of varying membrane thickness on the rate of He^+^ ion-facilitated nanopore expansion has so far not been studied. This aspect of the fabrication process is of central importance, chiefly because the HIM method does not allow *in situ* imaging of the ultimate device without imparting significant damage–a phenomenon that will affect ultrathin nanopore devices even more than conventional SS-nanopores. Therefore, a complete description of thickness dependence is necessary in order to realize arbitrary device dimensions. In this paper, we study the effect of membrane thickness on HIM nanopore expansion systematically. We first use lithographic ion exposure to reduce membrane thickness across a defined region (500 nm × 500 nm) of a suspended film and then form SS-nanopores in the patterned region (Figure 1). Through direct imaging of these pores, we observe a trend that can be used to predict the necessary He^+^ ion exposure to realize devices of any size, down to ∼3 nm in diameter and less than 2 nm in thickness.

## 2. Experimental Section

### 2.1. Fabrication & Characterization

Silicon chips, each supporting a free-standing, low-stress amorphous SiN membrane are purchased commercially (Protochips, Raleigh, NC, USA) and used as delivered. Ellipsometry measurements are performed to determine initial membrane thickness, *T_0_*. Prior to fabrication, chips are cleaned with acetone and ethanol and dried under a Nitrogen stream. The chips are then exposed to oxygen plasma (150 W) for 2 min before being loaded into a helium ion microscope (Zeiss Orion Plus, Carl Zeiss, Peabody, MA, USA). An additional treatment with air plasma (10 W, 3 min) is performed in the antechamber before samples are moved into the main chamber of the microscope. The ion beam current is adjusted initially to 5–6 pA through a 20 μm aperture with an accelerating voltage of 25 kV. We note that beam current is monitored to within 0.1 pA and that this value is used to calculate delivered dose actively. Beam shape and focus are optimized at a spot proximal to the suspended SiN membrane directly prior to membrane exposure with user-defined patterns. In order to reduce membrane thickness locally, defined regions of the as-purchased SiN membrane (original thickness *T_0_* = 18.2 ± 0.6 nm) are exposed lithographically using the focused beam of the HIM rastered over a 500 nm × 500 nm square pattern. Each (1 nm × 1 nm) point in the pattern is exposed to the ion beam for a brief time (0.1 μs) and multiple repetitions of the pattern are used to achieve the desired total ion dose. Under these conditions and for a thin membrane, we do not observe swelling effects or ion pressure-induced deformation of the surrounding membrane material [38]. Membrane thickness determination is then performed using an approach established in our previous work [39]. Briefly, patterns are exposed with various ion doses in a SiN membrane and the array of patterns is then imaged with scanning transmission ion microscopy using a custom holder. Analysis of these images [39] can be used to determine remaining membrane thickness.

Directly following the manipulation of local membrane thickness, a computer-controlled exposure at a single position is performed in order to produce an individual nanopore. The size of the exposed area is set by the focal diameter of the He^+^ beam. We have shown that the diameter of a nanopore fabricated in this manner is determined by the total time of exposure, and therefore by the total ion flux impacting the membrane [37]. Transmission electron microscopy is performed with a Carl Zeiss Libra 120. The total area of each nanopore is measured directly from the acquired images [40] and an average diameter, *d_p_*, is calculated assuming a perfectly circular pore. A low accelerating voltage (120 keV) is used in order to avoid modification by the beam itself.

### 2.2. DNA Translocation Measurements

SS-nanopore devices are first treated with oxygen plasma (150 W for 2 min) before being loaded into a custom Ultem 1000 flow cell. An electrolyte solution containing 900 mM NaCl and 10 mM Tris-EDTA (pH 8.0) is then introduced to both sides of a chip and an Ag/AgCl electrode is immersed in each reservoir to apply voltage and record electrical signals through the nanopore using a patch-clamp amplifier (Axopatch 200B, Axon Instruments, Foster City, CA, USA). Current-voltage characteristics are measured and stability is assessed with each device. The devices presented here demonstrate low noise (<30 pA) and a stable baseline current throughout the measurement. 3 kbp dsDNA (10 ng/μL) is then introduced to one chamber and a trans-membrane voltage of 300 mV is applied. Conductance blockade events are collected at 200 kHz and the electrical signal is subjected to a 100 kHz four-pole Bessel filter.

## 3. Results and Discussion

### 3.1. Ultrathin Nanopore Formation & Characterization

**Figure 2.**
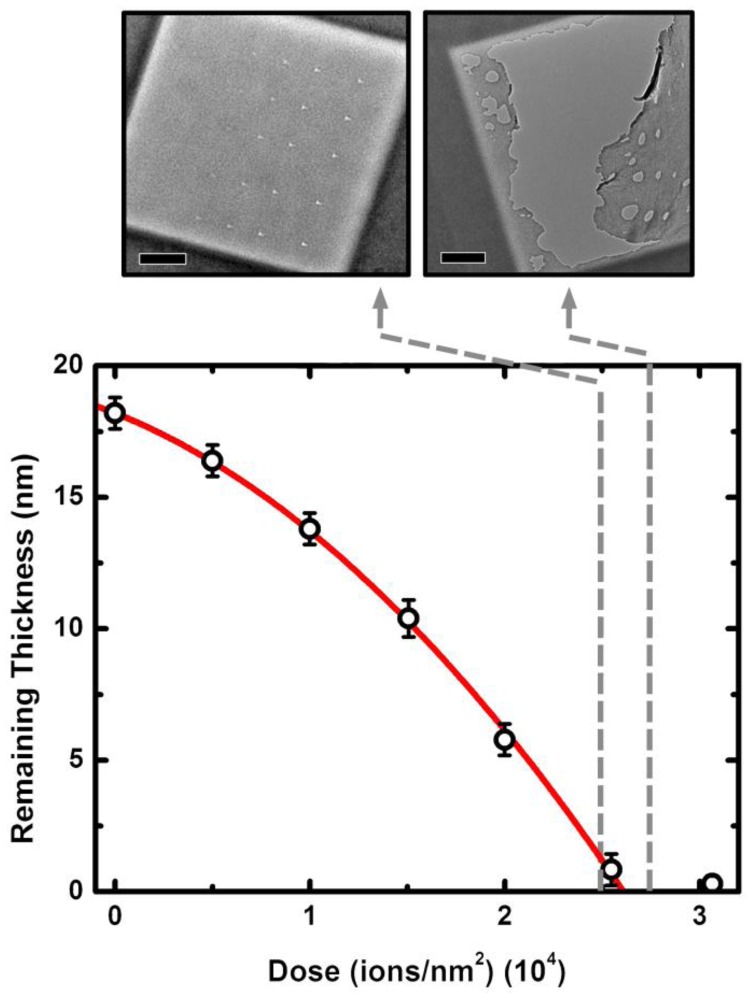
Membrane thinning analysis. Relationship between total He+ ion dose and remaining thickness of a SiN membrane (initial thickness 18.2 nm), as determined by transmission Helium ion analysis. Solid line is a second-order polynomial fit to the data. Each dashed arrow leads to a transmission electron micrograph of a 500 nm × 500 nm patterned region milled with the indicated dose (2.50 (**L**) and 2.75 (**R**) ×10^4^ ions/nm^2^). A dot array is formed within the pattern as a marker to indicate whether the local membrane remains. Scale bars are 100 nm.

Analyzing the relationship between membrane thickness and He^+^ ion exposure dose (Figure 2), we find a trend that can be described well by a second order polynomial, similar to previous results [38,39]. In order to confirm the precision and repeatability of our fabrication technique, we also form patterns on a separate chip from the same wafer using exposure doses in a narrow range surrounding the “breakthrough” dose (*i.e.*, the ion dose resulting in complete removal of the membrane, 2.60 × 10^4^ ions/nm^2^). Subsequent transmission electron microscopy verifies that a dose only slightly less than the point of breakthrough (2.50 × 10^4^ ions/nm^2^) results in a continuous film at the patterned region, while a slightly higher dose (2.75 × 10^4^ ions/nm^2^) results in a broken membrane (Figure 2, top). Consequently, the ion dose-thickness relationship can be used to tailor the dose needed to achieve a reduced local membrane thickness, *T_t_*, of nearly any desired value.

If we assume a SiN material density of 3.4 g/cm^3^ and a molecular weight of 140.3 g/mol, we can use the remaining membrane thickness to calculate the milling yield, *S*, for the system, defined as the ratio of sputtered atoms to ions impinging on the substrate. We find a mean value of 0.008 and note that the value rises as high as 0.01 due to increasing transmission milling of the membrane [38,39]. This value is in reasonable agreement with simulation results [39,41] that yield a value for *S* of 0.02 (data not shown). The discrepancy may be the result of redeposition of sputtered material [42] or differences in material properties (density, *etc.*) that can change significantly for different membrane growth processes. While little variation is observed in the milling behavior of membranes from the same batch, this highlights the fact that membranes of different solid-state material or even those formed under slightly different conditions would require independent analysis in order to characterize the ion dose-thickness relationship properly.

**Figure 3.**
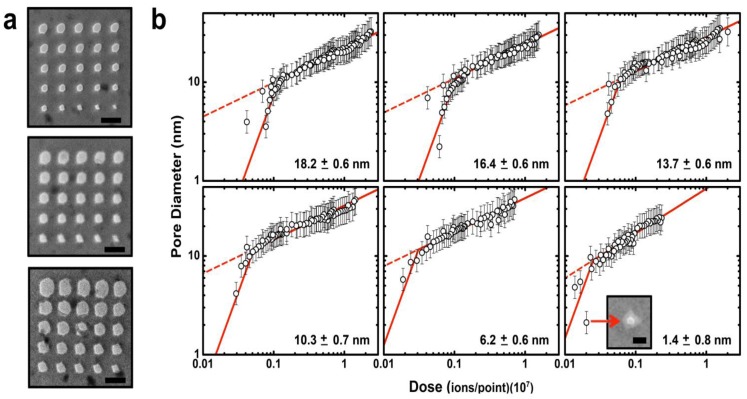
Solid-state nanopore expansion as a function of membrane thickness. (**a**) Transmission electron micrographs of three nanopore arrays formed in different membrane thicknesses (T-B: 18.2 ± 0.6, 13.7 ± 0.6, and 6.2 ± 0.6 nm) with the same range of He+ ion doses (0.4 − 7.1 × 10^6^ ions/point). Scale bars are 50 nm; (**b**) Log-log plots of ion dose *vs.* resulting pore diameter over a range of membrane thicknesses, indicated in the lower right of each plot. Solid lines are power law fits to the two sections of the data. Dashed lines are continuations of the lower slope (right side) fit, illustrating the deviation from that trend at low dose. Inset (lower right) shows a transmission electron micrograph of the smallest pore (∼2 nm diameter) achieved in the thinnest membrane. Scale bar is 5 nm.

Following membrane thinning, individual pores can be formed and characterized within the patterned regions (Figure 3a). In previous work [37], we have found that He^+^ ion milling of SS-nanopores proceeds via a two-regime process, with a fast expansion rate observed at low ion dose and a slow rate observed at higher ion dose. We have proposed that these two regimes are due to the Gaussian intensity profile of the ion beam. For brief exposure times (low dose), the intense center of the beam contributes strongly to the milling process, causing rapid pore expansion. However, for long exposure times (high dose), the center of the beam passes through the milled opening of the pore, thus allowing only the less intense outer beam to contribute to material removal and reducing the expansion rate. In Figure 3b, we show that a similar trend is observed across a range of average thickness from 18.2 ± 0.6 down to 1.4 ± 0.8 nm. Interestingly, we find that the transition point between the fast and slow expansion regimes occurs for all membrane thicknesses at a nanopore diameter of ∼10 nm. This is consistent with our past measurements [37] and with our proposed mechanism, since the transition would be determined by the diameter of the beam rather than characteristics of the substrate.

As shown in Figure 3b, we find that the slopes of power-law fits to the data on a log-log scale are quantitatively reproducible across all investigated membrane thicknesses. We note that the second (slower) expansion regime for *T_t_* = 1.4 ± 0.8 nm is slightly steeper than in other data sets, but this may be caused by a minor systematic variation in beam current or possibly by material rearrangement due to surface energy minimization [43]. Importantly, as the membrane thickness is decreased, we find that the observed trends are shifted to lower ion doses, indicating that the same ion exposure will result in a pore of larger diameter. This is again in agreement with our model for the two expansion regimes [37]: given a constant He+ beam diameter, a thin membrane should require less ion dose to achieve the same nanopore size due to the reduction in material volume that must be removed. Future experiments may be able to explore this in more detail by, for example, varying He^+^ ion beam focal diameter. Still, we note that this method can produce nanopores as small as ∼2 nm with these conditions (Figure 3b, inset). SS-nanopores are difficult to form in membranes thinner than 1.4 ± 0.8 nm without inducing wider damage in the frail surrounding membrane.

**Figure 4.**
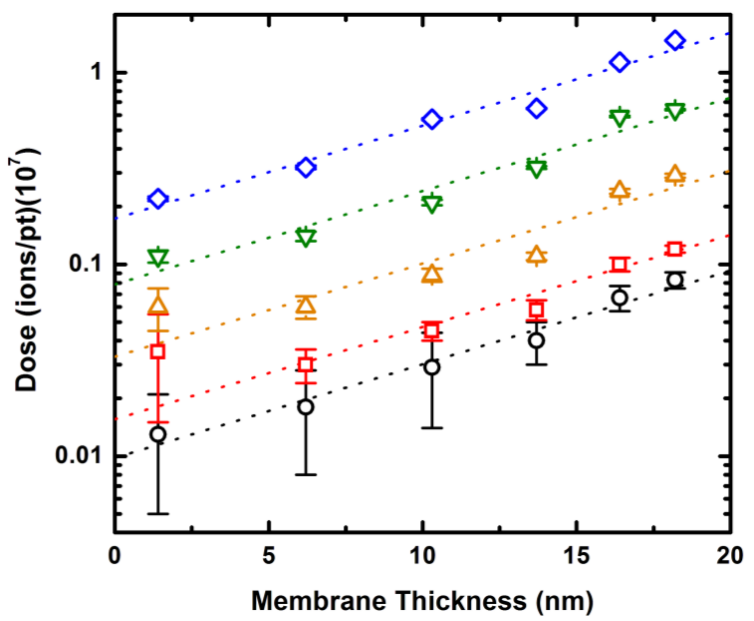
Pore expansion dynamics vary predictably with membrane thickness. The dose required to achieve a target nanopore diameter plotted as a function of membrane thickness. The datasets are for pore diameters (from bottom to top) of 5 (black circles), 10 (red squares), 15 (orange upward triangles), 20 (green downward triangles), and 25 (blue diamonds) nm, respectively. The dose required for all representative pore diameters varies as a monoexponential with membrane thickness. The slopes agree within less than 1%.

By understanding how the trends observed in Figure 3b depend on membrane thickness, the exposure conditions required to achieve a wide range of device dimensions can be predicted. Because of the reproducible shape of those trends, this amounts to a quantitative description of how the He^+^ ion dose associated with any particular SS-nanopore diameter shifts with membrane thickness. In order to explore this, we consider target diameters from 5 to 25 nm as characteristic examples of the trend. As can be seen in Figure 4, we find that the required dose for each individual pore diameter rises with a monoexponential dependence as membrane thickness increases. Importantly, the exponents of these dependencies (*i.e.*, the slope of the semi-log plots) are highly consistent, varying less than 1% across all data sets. As a result, it is therefore possible to interpolate the dose-diameter relationship for any membrane thickness. Coupling this fine control over diameter with the independent control over membrane thickness demonstrated above, we attain the ability to fabricate SS-nanopore devices with nearly arbitrary geometry reproducibly. We estimate that membrane thickness can be tailored to within ∼1 nm of a desired value and nanopore diameter can be controlled to within 2–3 nm, on average. These errors may be improved upon by, for example, increasing the accuracy of the He^+^ ion beam current measurement used to determine dose.

### 3.2. DNA Translocation Through an Ion-Thinned Nanopore

As a proof of principle to the utility of the presented approach, we next explore dsDNA translocations through our SS-nanopore devices. We fabricate two devices in membranes of different thickness: 4.5 ± 0.6 (thin) and 24.5 ± 0.8 nm (thick), respectively. We use the linear current-voltage characteristics of the open pores to determine device diameter geometrically, taking into consideration the access regions [27,44], which are increasingly important as device thickness is decreased. SS-nanopore diameter *d_p_* can be written as:

(1) $$d_{p}=\frac{1+\sqrt{\frac{16}{\pi }RT_{\text{eff}}\sigma_{\text{ion}}+1}}{2R\sigma_{\text{ion}}}$$

where *R* is the measured resistance of the nanopore and *T_eff_* is the effective thickness of the SiN membrane. Because the width of the He+ beam is large compared to the target pore diameter [37], the resulting nanopores are hourglass-shaped in cross-section rather than purely cylindrical. Thus, we use the convention of previous work [27] that established *T_eff_* = *T_t_/3* to account for the shape of the pore. The variable σ*_ion_* is equivalent to *n_i_e(μ_Cat_* + *μ_An_)*, where *n_i_* is the number density of ions in solution, *e* is the elementary charge, and *μ_Cat_* and *μ_An_* are the electrophoretic mobilities of the cation and anion, respectively. By measuring the resistance of the nanopore and applying its known thickness, we find that the nanopores presented here have diameters of 3.2 nm (thin membrane) and 3.1 nm (thick membrane), respectively. Thus the devices are nearly equal in diameter, allowing the effects of membrane thickness on measurement sensitivity to be isolated.

**Figure 5.**
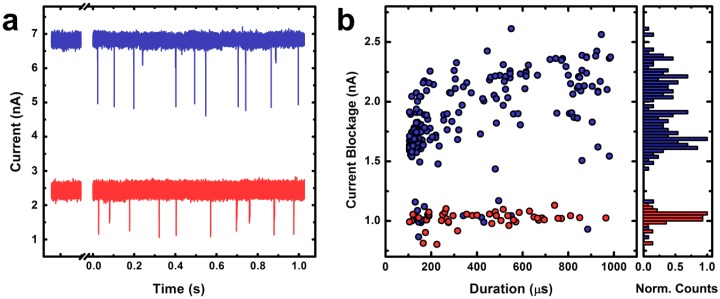
dsDNA translocation measurements. (**a**) Typical raw current traces measured with two SS-nanopore devices: one 3.2 nm in diameter in a 4.5 ± 0.6 nm thick membrane (blue) and one 3.1 nm in diameter in a 24.5 ± 0.8 nm thick membrane (red). Upon addition of dsDNA, a series of translocation events are recorded. Traces are subject to a low-pass filter of 10 kHz; (**b**) Scatter plot of event durations and mean current blockages for events recorded through the thin (blue, *n* = 191) and thick (red, *n* = 56) devices in (a). Accompanying current blockage histogram shows significantly (∼2-fold) deeper events are observed for the thin SS-nanopore device.

Following device characterization, linear 3 Kbp dsDNA in measurement solution is introduced to the electrically-grounded reservoir of the flow cell. A trans-membrane voltage of +300 mV is applied to the opposite electrode and the resulting ionic current signal is collected at 200 kHz before being low-pass filtered at 10 kHz. Figure 5a shows typical raw current traces for the thin (blue) and thick (red) devices, respectively. The displayed baseline currents are not shifted for clarity, but instead are well-separated due to device geometry. In both cases, a series of downward spikes (events) are observed. Immediately, it can be seen that the thin device yields events that are deeper (larger change in current, Δ*I*) in comparison to those observed in the thick device for the same experimental conditions. The data in Figure 5b makes this clear, showing an analysis of average current blockage for events through each device. Here, only events corresponding to molecular translocations are considered, ignoring shallow events that have been attributed to “collisions” with the pore [45–47]. Considering that the noise level in both devices was roughly equivalent (25–30 pA RMS), the average depth (signal) of events can be compared between the two systems to indicate the SNR enhancement afforded by membrane thinning. We find, on average, about a two-fold increase in SNR for the thin device. We note that this factor can be increased further in alternative measurement solutions [27].

## 4. Conclusions/Outlook

In conclusion, we have shown that the beam of a helium ion microscope can be used to customize both the thickness and diameter of SS-nanopores formed in free-standing SiN membranes, achieving reproducible dimensional control of devices at the nanometer scale. From transmission HIM images, we were able to characterize the control of membrane thickness using He^+^ ion exposure. We investigated the ion dose-diameter relationship of SS-nanopores fabricated in membranes with various thicknesses using TEM measurements. We demonstrated that a quantitatively reproducible trend in nanopore expansion is found for all studied thicknesses down to 1.2 ± 0.8 nm. Importantly, we established that this reproducible trend shifts with membrane thickness monoexponentially. Thus, our data can be extrapolated to any membrane thickness based on a single calibration, enabling arbitrary control over device dimensions. These results demonstrate that HIM milling can serve as a highly flexible fabrication process within the limits of the system; we have shown here that this technique can produce nanopores as small as 2 nm in diameter within a membrane as thin as 1.2 ± 0.8 nm. Individual devices with target geometries were achieved and studied to demonstrate the efficacy of SS-nanopores formed with our method. By translocating dsDNA through two SS-nanopores with nearly the same diameter (3.2 and 3.1 nm) but with different membrane thicknesses (4.5 ± 0.6 and 24.5 ± 0.8 nm), we showed that a two-fold increase in SNR can be achieved through membrane thinning for our experimental conditions.

This method enables flexible fabrication of ultrathin SS-nanopores without combining multiple techniques or instruments. Our approach is rapid, taking less than 1 min, even for the thinnest membrane, and offers a level of control uncommon to other fabrication techniques. This technique will allow for optimized production of SS-nanopores with increased sensing resolution for small molecules or molecular features.
